# National antibiotic consumption for human use in Chad (2017–2021): a descriptive cross-sectional study

**DOI:** 10.3389/frabi.2025.1612557

**Published:** 2025-07-11

**Authors:** Zongo R. Frank Edgard, Kadidjia Bakari Traoré, Colette Ngabéré, Martine Yoyammel, Abatcha Oumar Kadai, John Eyong Efobi, Mathieu Hota, Didi Lamireou, Badawi Haroun Mahamat, Hamit Mahamat Alio, Jacques L. Tamuzi, Patrick D. M. C. Katoto, Charles S. Wiysonge, Blanche-Philomene Melanga Anya

**Affiliations:** ^1^ Organisation Mondiale de la Santé, N’djamena, Chad; ^2^ Centrale Pharmaceutique d’Achats, Ministère de la santé publique, N’djamena, Chad; ^3^ Direction Générale de la pharmacie, du médicament et des laboratoires, Ministère de la santé publique, N’djamena, Chad; ^4^ Institut de Recherche en Elevage pour le Développement (IRED), N’djamena, Chad; ^5^ Université de N’djamena, N’djamena, Chad; ^6^ Division of Epidemiology and Biostatistics, Department of Global Health, Faculty of Medicine and Health Sciences, Stellenbosch University, Cape Town, South Africa; ^7^ Centre for Tropical Diseases and Global Health, Department of Medicine, Catholic University of Bukavu, Bukavu, Democratic Republic of Congo; ^8^ Cochrane South Africa, South African Medical Research Council, Cape Town, South Africa; ^9^ World Health Organization Regional Office for Africa, Brazzaville, Democratic Republic of Congo

**Keywords:** ABC, AWaRe, MRC, DDD, ABR, Chad

## Abstract

**Background:**

Antibiotic resistance (ABR) to commonly used antibiotics is significant in sub-Saharan Africa (SSA). In SSA, Chad has one of the highest antimicrobial resistance (AMR) rates. The link between ABR and antibiotic consumption (ABC) is well-established. However, no ABC-related studies have been conducted in Chad recently. The purpose of this study is to examine the trajectory of ABC in Chad from 2017 to 2021, using the World Health Organization’s (WHO) Access, Watch, and Reserve (AWaRe) antibiotic classification.

**Methods:**

A descriptive retrospective study was conducted in N’Djamena, using antibiotic import and distribution data collected from the General Directorate of Pharmacy and four wholesale distributors of medicines. The defined daily doses (DDDs) and the mean relative change (MRC) were used to compute the results. Results were presented in terms of tables and graphs. The results were compared to the WHO’s guidelines for ABC use via the AWaRe categorization.

**Results:**

Between 2017 and 2021, an average ABC of 2.5 doses per inhabitant per year was observed, peaking in 2020 at 5.3 doses per inhabitant. In terms of DDD, the ten most commonly consumed antibiotics during the time, in descending order, were amoxicillin, ampicillin, sulfamethoxazole/trimethoprim, doxycycline, ciprofloxacin, phenoxymethyl-penicillin, erythromycin, ceftriaxone, azithromycin, and gentamicin. However, the MRC analysis detected an increase in benzathine benzyl penicillin, benzylpenicillin, ampicillin, amoxicillin+clavulanic acid, flucloxacillin, ceftriaxone, cefixime, cefpodoxime and cefalexin, cotrimoxazole, ciprofloxacin, levofloxacin, norfloxacin, ofloxacin, and azithromycin. Controversially, amoxicillin, cefotaxime, doxycycline, erythromycin, and moxifloxacin had a lower MRC from 2017 to 2021. Although 90% of the ABC are from the “Access” group, the “Watch” group has increased over time.

**Conclusions:**

Our findings indicated a significant ABC in the Chadian population from 2017 to 2021, which may elucidate the country’s elevated ABR. On average, 90% of ABC were categorized in the “Access” group, although utilization of the Watch group increased over time. This requires the prompt implementation of the monitoring system for ABC at all tiers in Chad.

## 1 Introduction

In 2021, bacterial antibiotic resistance (ABR) accounted for 4.71 million deaths, with 1.14 million directly attributable to it (Naghavi, 2024). By 2050, ABR may cause 1.91 million direct deaths and 8.22 million associated deaths globally (Naghavi, 2024). ABR is a major threat to human development as it affects our ability to treat a range of infections caused by bacteria, parasites, viruses, and fungi (World Health Organization, 2018). Modern medical procedures, such as major surgery, organ transplantation, treatment of preterm babies, diabetes management, and cancer chemotherapy, will become very high risk without effective antibiotics (Laxminarayan et al., 2013; WHO, 2018; World Health Organization, 2018). ABR is identified by the World Health Organization (WHO) as one of the most serious threats to global public health (Maugat et al., 2019). A study revealed a high level of ABR to the commonly used antibiotics in the sub-Saharan African (SSA) region (Tadesse et al., 2017). In SSA, this problem is further aggravated by other factors such as the over-the-counter sale of antibiotics, self-medication, and unauthorized use of antibiotics. A systematic review revealed a positive association between ABR and antibiotic consumption (ABC), both in community and hospital settings (Abejew et al., 2024). Furthermore, Holmes et al. demonstrated that mass drug administration for human health is a potential driver of ABR (Holmes et al., 2016). Except for some antibiotics, increased ABC is associated with increased ABR and decreased ABC is associated with decreased ABR (Abejew et al., 2024). The available evidence suggests that the global ABC in humans has risen in the past two decades, primarily driven by an increased use in low- and middle-income countries (Van Boeckel et al., 2014; Klein et al., 2018). At the same time, there has been a shift toward the use of broad-spectrum and last-resort antibiotics (Klein et al., 2018). These trends are partly a result of improved access to medicines because of economic development in some parts of the world, but also because antibiotics are used inappropriately. The patterns of inappropriate use of antimicrobials include the use of antibiotics to treat conditions that are not caused by a bacterial infection, the use of the wrong type of antibiotic, the use of the wrong dosage or route of administration, and use for the wrong duration. Detailed global estimates are lacking, but in countries that are part of the Organization for Economic Co-operation and Development (OECD), as many as half of all antimicrobials used in human health care can be considered inappropriate (Cecchini, 2015; World Health Organization, 2018). At the sixty-eighth World Health Assembly held in May 2015 in Geneva, WHO member countries adopted the Global Action Plan to Combat ABR. The overall goal of the plan was to maintain, for as long as possible and without interruption, the capacity to treat and prevent infectious diseases using safe, effective, and quality-assured medicines and enable the responsible use and accessibility of antimicrobials to all those who need them (WHO, 2026). The Access, Watch, and Reserve (AWaRe) antibiotic classification system is a valuable tool for tracking ABC, setting performance targets, and evaluating the effects of the antimicrobial stewardship program (ASP), aiming to optimize antibiotic use and limit ABR development (WHO, 2021a; Mthombeni et al., 2022).

However, little is known about AWaRe in the African region and particularly in Chad. A study highlighted that there was a poor representation of data from Africa in the AWaRe analysis conducted at a global level (Klein et al., 2021). In Chad, a study indicated that it is imperative and essential to execute awareness-raising initiatives for the population and prescribers regarding rational ABC to help curtail the proliferation of resistance strains (Ndoutamia et al., 2014). In 2019, Chad ranked in the top 10 nations globally for age-standardized mortality rates linked to ABR (Cardinale et al., 2020). Research indicated that the indigenous bacterial population in N’Djamena exhibited significant resistance to widely utilized antibiotics (Cardinale et al., 2020). Despite lacking legal authorization to provide medications, illicit drug vendors deliberately establish their operations in proximity to both public and private healthcare facilities in Chad. Presently, items sold in the country require a Marketing Authorization (MA) for each antibiotic; yet, their traceability poses challenges for both supply and dispensing (Ministere de la Sante Publique, 2018). The rampant sale of illegal antibiotics in markets and streets, together with their unregulated distribution in pharmacies and warehouses, exacerbates the issue of ABR in Chad (Ministere de la Sante Publique, 2018).

**Figure 1:**
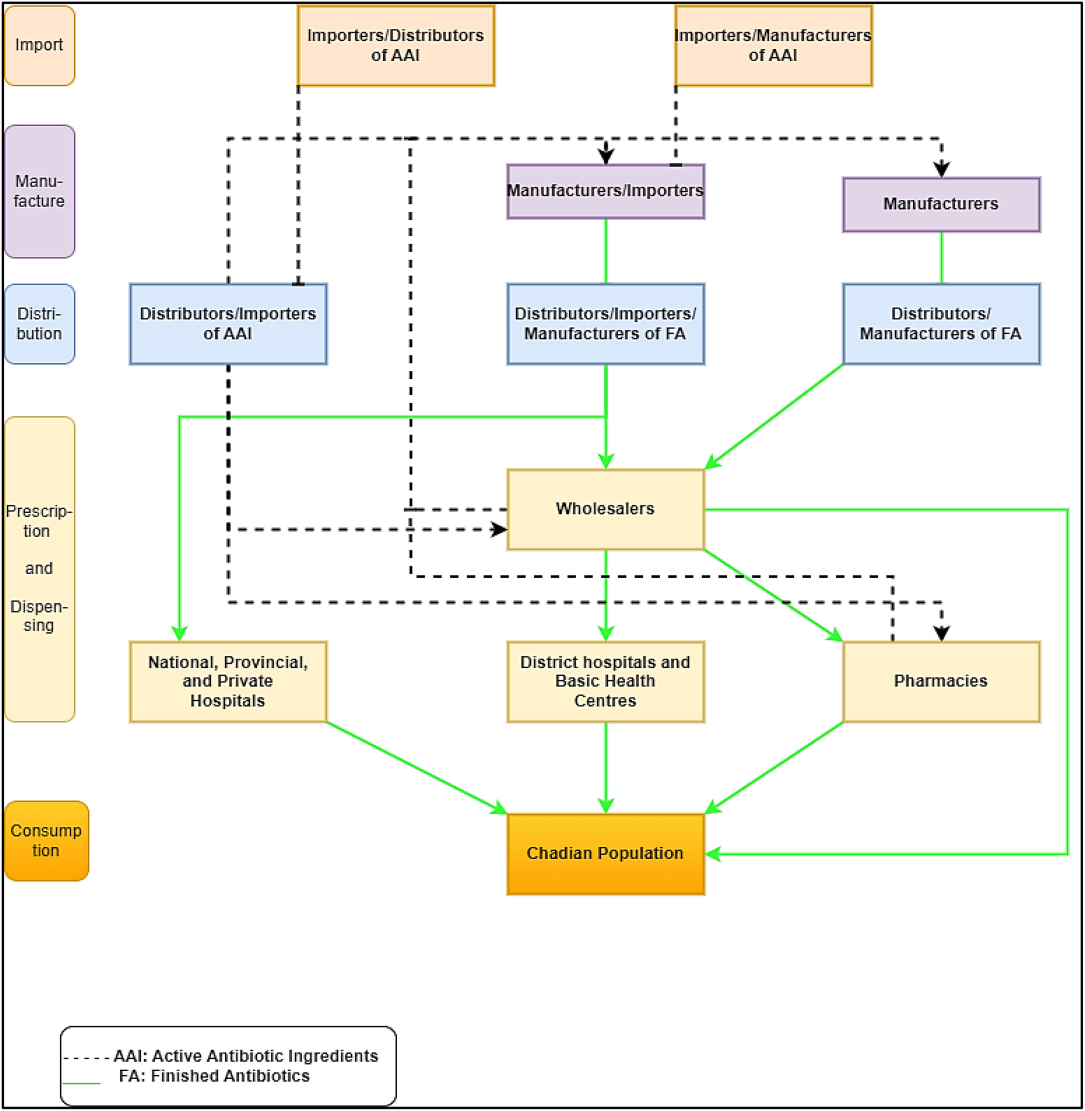
System adapted and integrated for the commercial manufacture of antibiotics in Chad.

Therefore, the literature has shown that there has been no AWaRe study undertaken in Chad recently. One of the effective means for this prevention is to set up a system to monitor ABC in Chad. This will allow the collection of reliable statistical data to present the trends. Considering the characteristics of pharmaceutical statistics, a direct comparison should be approached with caution. **Figure 1** shows the ABC flow and its components in Chad. Since the growth in pharmaceutical spending has greatly increased over the past decade, ABC needs to be monitored. Health statistics of pharmaceutical use and expenditure are essential to make and implement evidence-based pharmaceutical policy (Kim et al., 2020). This study aims to investigate the methods and results of ABC and sales according to the sources and assess the trend of antibiotic use from 2017 to 2021 in Chad.

## 2 Methods

### 2.1 Study design

This was a descriptive and cross-sectional study of ABC data estimated from import and wholesale distribution in Chad from 2017 to 2021.

### 2.2 Study setting

Chad is a landlocked nation, with the closest harbor, Douala in Cameroon, located approximately 1,000 miles distant. This isolation can substantially affect the availability of pharmaceuticals. The population of Chad is 15,778,417 individuals. The population is widely spread across the land, with an average density of 10.6 persons per km². In Chad, poverty and vulnerability are prevalent, with 44.8% of the population residing below the national poverty threshold in 2022. Extreme poverty ($2.15/day per capita [2017 PPP]) rose by 2.6 percentage points from 2023 to 2024, reaching 36.5% (World Bank, 2024). This rise indicates that 688,000 more individuals have descended into extreme poverty (World Bank, 2024). Additionally, there are significant regional disparities in the distribution of poverty. Social inequalities have also slightly increased, as evidenced by the Gini index (World Bank, 2024).

In Chad, the pharmaceutical industry has (i) a regulatory structure in the form of the General Directorate of Pharmacy, Medicines, and Laboratories (DGPML), which serves as the National Pharmaceutical Regulatory Authority (ANRP), with a national medicine commission in charge of approval and a committee of pharmacovigilance experts, and (ii) supply and distribution structures. Public supply institutions include the Central Pharmaceutical Purchasing Office (CPA) and 23 Provincial Supply Pharmacies (PPAs), which are currently self-sufficient and associated with the country’s 23 provincial health delegations. At the private level, the most recent establishment mapping for 2023 indicates 50 pharmacies, including 47 in N’djamena; 205 pharmaceutical depots, including 147 in N’djamena; and 60 pharmaceutical wholesalers, including 57 in N’djamena. Less than 10 of these private wholesalers are still operational and active. The country has no manufacturers. It is also worth noting that a Logistics Management Unit (LMU) is in charge of monitoring availability and gathering logistics data, as is a national supply coordination commission with quantitative subcommittees. The pharmaceutical regulatory system is in its early stages (level 1 on a 1–4 scale), and the ANRP lacks autonomy and independence.

### 2.3 Inclusion and exclusion criteria

The study included all antibiotics imported and distributed by wholesale structures certified by the Ministry of Health as part of the legal and licit drug circuit. Antibiotics imported and distributed by structures that refused to participate were excluded.

### 2.4 Outcomes and definitions

- Full designation in the international common name (INN) of the antibiotic.
- Number of commercial antibiotic designs assessed.
- Number of common international antibiotic designations assessed.
- Defined daily dose (DDD): The DDD is the expected daily maintenance dose for a medication used for its primary indication in adults. The DDDs per 1,000 persons per day can be roughly translated as the average number of people per 1,000 inhabitants receiving antibiotic therapy each day (WHO, 2024a).
- AWaRe classification of each antibiotic, including (i) the ACCESS group (A), which includes antibiotics that are effective against a wide range of regularly encountered susceptible infections while having a lesser resistance potential than antibiotics in other groups (Zanichelli et al., 2023) and can be used as either first- or second-line treatment; and (ii) the WATCH group (Wa), which represents antibiotics that are more prone to toxicity and have a higher risk of developing resistance (Zanichelli et al., 2023). These antibiotics are only advised for specialized and limited uses (Zanichelli et al., 2023). The usage of these antibiotics should be closely monitored by snapshot prevalence studies (Zanichelli et al., 2023).

### 2.5 Data collection and management

Our input data consisted of the number of international commercial and common designations of antibiotics. The quantity of antibiotics dispensed was extracted using the CPA’s inventory management software, DGPML import database, Central Pharmaceutical Purchasing Office database, and wholesalers’ stock management database. Data were collected using an Excel spreadsheet including the number of commercial and common designations of antibiotics recorded (import vs. wholesale data), including the code ATC, year, pharmacological classification of antibiotics (penicillin, other beta-lactam antibacterials, quinolones, macrolides, tetracyclines, chloramphenicol, sulfonamides and trimethoprim, aminoglycosides, combination of antibacterials, and other antibacterials), and ABC in DDDs. Data analysis included comparing the overall ABC over the years and according to the AWaRe classification. For each antibiotic, we collected the number of pharmaceutical units and the number of DDDs per 1,000 inhabitants/year. The revalidation process, management, and cleaning to simplify the data were done using Stata statistical software (Version 18 MP).

### 2.6 Data analysis

The DDD for each antibiotic agent was compiled to ascertain the total DDDs at the specified ATC level and adjusted for population size, utilizing data from Chad to calculate the absolute consumption rate in DDDs according to the equation. For cross-country comparisons, the metric “defined daily doses per 1,000 inhabitants per day” was employed to report antibiotic consumption in both import and wholesale settings (in alignment with the WHO Anatomical Therapeutic Chemical classification), offering a general estimate of the proportion of the population receiving antimicrobials daily. A descriptive analysis was conducted, utilizing means, standard deviations, and distributions to characterize numerical variables. A central tendency test was conducted by sorting the data in ascending order, determining the median, and selecting the drug with a higher mean relative change (MRC) above the median to illustrate the trends in the increase and decline in antibiotic consumption. The MRC in percentage terms was determined using the first year as a standard and the relative change 4 years from the original standard year (Laxminarayan et al., 2016). The DID equation determines the quantity of DDDs utilized by 1,000 patients daily. The derived value was delineated as follows: for every 1,000 patients, a specific number of DDDs are utilized daily. Each antibiotic was categorized into AWaRe classifications according to the most recent edition of the WHO model list of essential medicines (Institute for Health Metrics and Evaluation, 2024). Antibiotic compounds that do not appear on the WHO Model List of Essential Medicines are classified as “Other” (WHO, 2021b). Reported metrics and data analysis are illustrated in Equations 1–3.


(1) $$\begin{array}{c} DID=\;\\ \frac{Consumption\;of\;a\;specific\;medicine\;or\;subgroup\;in\;DDDs}{\left(Number\;of\;inhabitants\right)\times \;(Number\;of\;days\;in\;periods\;of\;data\;collection\;)}\times 100 \end{array}$$

(2) $$\begin{array}{c} Access-to-Watch\;Index\\ =\;\frac{Number\;of\;DID\;of\;Access\;antibiotics\;}{Number\;of\;DID\;of\;Watch\;antibiotics\;} \end{array}$$

(3) $$\begin{array}{c} Mean\;Relative\;Change\\ =100\;\times \;\frac{DDD\;final\;Value-DDD\;initial\;Value}{\left[DDD\;initial\;Value\right]} \end{array}$$

### 2.7 Ethical approval

This study was reviewed and approved by the Ministry of Health of Chad. We have also received authorization from the Dean of the Faculty of Human Health Sciences (FSSH), the Director of the CPA, the Director General of the DGPML, and the Directors General of the wholesale distributors. This study was conducted in accordance with the Helsinki Declaration and local institutional policies on human research. Furthermore, the data were collected anonymously, and the names of the wholesale structures are not included in the tools or results.

## 3 Results

### 3.1 Daily dose consumption by pharmacological groups from import and wholesale data

**Figure 2:**
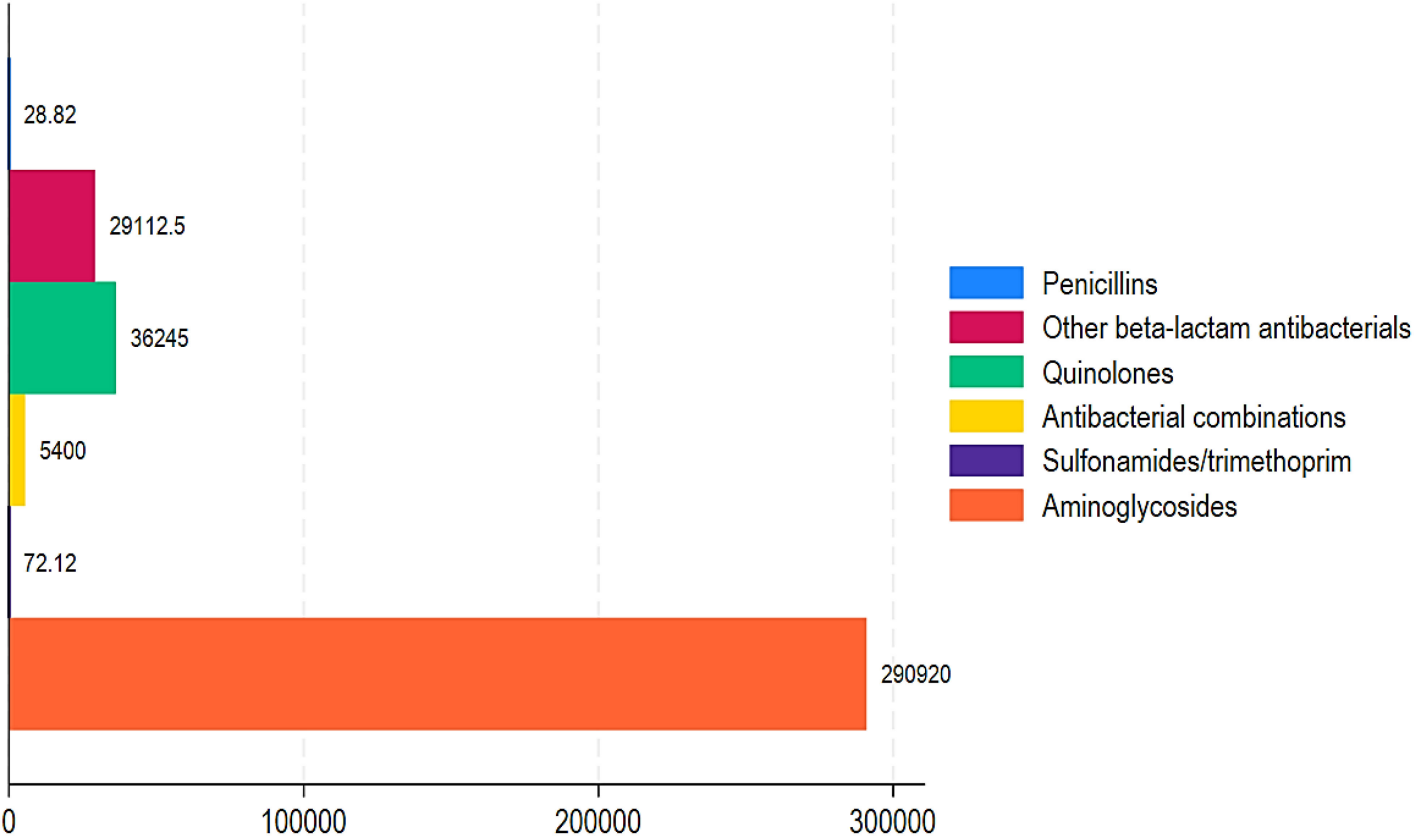
Proportion of positive mean relative change in mean DDD in 1,000 inhabitants annually in Chad from 2017 to 2021.

**Figure 3:**
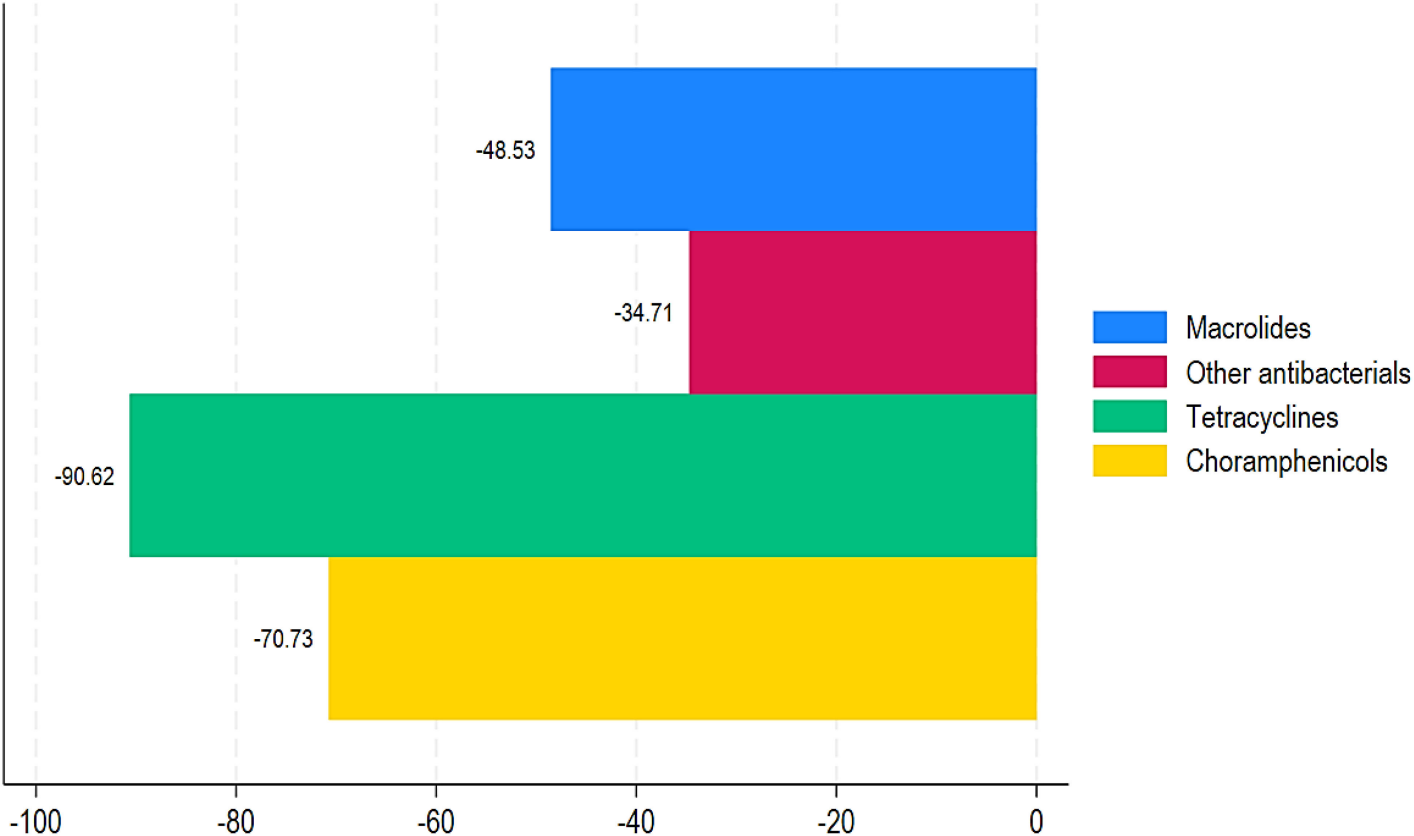
Proportion of negative mean relative change in mean DDD in 1,000 inhabitants annually in Chad from 2017 to 2021.

**Figure 4:**
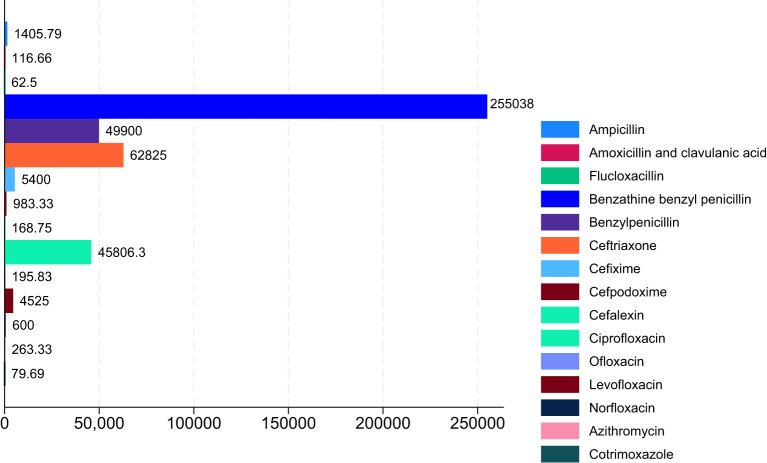
Proportion of positive mean relative change in ABC in Chad (2017–2021).

**Table 1** displays the number of antibiotic trade names and international common antibiotic names recorded per year using import and wholesale distribution statistics, respectively. Imported data contained an average of 139 antibiotic trade names and 32 worldwide common antibiotic names. **Tables 2**, **3** show the ABC data estimated from import and distribution data from 2017 to 2021. It varies between 23 million doses and approximately 87 million for import data and between 23 million doses and approximately 68 million for wholesale data. There is a peak in ABC in 2020, regardless of the data source and an average of approximately 42.5 million doses of antibiotics consumed each year. From wholesale data, the ABC of pharmacological groups during the 5 years (2017 to 2021) in descending order is as follows: penicillin with more than 128 million doses (with ABC reaching a peak of more than 57 million doses in 2020), sulfonamides/trimethoprim with 17.5 million doses, cyclins with 11.9 million doses, quinolones with 11.8 million doses, macrolides with 9.6 million doses, and cephalosporins with 4.2 million doses (**Figure 2**). Consumption of the other groups is less than 1 million doses over 5 years. The ABC trend was quite similar when considering import data and wholesale data. However, the trend in ABC by DDDs per 1,000 inhabitants and MRC (%) from 2017 to 2021 for penicillin and sulfonamides/trimethoprim was a slight increase of 39.08% and 84.50%, respectively. The MRC (%) of aminoglycosides, other beta-lactam antibacterials, quinolones, and antibacterial combinations showed high increases of 36,2174.25%, 50,544.21%, 41,044.47%, and 6383.54%, respectively (**Table 2**; **Figure 3**). In contrast, tetracyclines, chloramphenicols, macrolides, and other antibacterials showed a decrease in ABC from 2017 to 2021 with MRCs of -90.94%, -69.75%, -44.50%, and -30.98%, respectively (**Table 2**, **Figure 4**).

**Table 1:** ABC by pharmacological group expressed in DDDs per 1,000 inhabitants and MRC.

Code ATC	Pharmacological groups	2017	2018	2019	2020	2021	Total mean relative change (%)
J01C	**Penicillins**	1,318.6	1,647.5	795.8	3,574.4	776.7	8,113
Mean relative change (%)			24.94	-39.65	171.07	-41.09	28.82
J01D	**Other beta-lactam antibacterials**	0.2	3.1	7.4	62.5	160.7	233.9
Mean relative change (%)			1,450	3,600	31,150	80,250	29,112.5
J01M	**Quinolones**	0.5	12.7	304.3	223.4	186.5	727.4
Mean relative change (%)			2,440	60,760	44,580	37,200	36,245
J01F	**Macrolides**	202.5	128.2	6.3	236.8	45.6	619.4
Mean relative change (%)			-36.69	-96.89	16.94	-77.48	-48.53
J01X	**Other antibacterials**	80.3	126	8.2	73.5	2	290
Mean relative change (%)			56.91	-89.79	-8.47	-97.51	-34.71
J01R	**Combination of antibacterials**	0.02	0.5	0.7	0.7	2.5	4.42
Mean relative change (%)			2,400	3,400	3,400	12,400	5,400
J01A	**Tetracyclines**	581.7	212.5	1.4	0.7	3.6	799.9
Mean relative change (%)			-63.47	-99.76	-99.87	-99.38	-90.62
J01B	**Choramphenicols**	12.3	11	2	0.7	0.7	26.7
Mean relative change (%)			-10.57	-83.74	-94.31	-94.31	-70.73
J01E	**Sulfonamides and trimethoprim**	141.5	305.5	387.1	17.1	264.5	668.7
Mean relative change (%)			115.90	173.57	-87.91	86.92	72.12
J01G	**Aminoglycosides**	0.005	0.004	1.3	1.7	55.2	58.2
Mean relative change (%)			-20	25900	33900	1103900	290920

**Table 2:** ABC expressed in DDDs per 1,000 in habitants annually and the associated MRC.

Antibiotics	Route	2017	2018	2019	2020	2021	Total mean relative change (%)
Penicillin							
**Amoxicillin**	Oral	1147.4	485.8	657.9	2,500.1	325.1	5,116.3
Mean relative change (%)			-57.66	-42.66	117.89	-71.67	-13.52
**Ampicillin**	IV	20.3	115.4	6.4	838.4	262.5	1243
Mean relative change (%)			468.47	-68.47	4,030.05	1,193.10	1,405.79
**Phenoxymethylpenicillin**	Oral	150.2	18.1	129.2	116.4	182.3	596.2
Mean relative change (%)			-87.95	-13.98	-22.50	21.37	-25.76
**Amoxicillin + clavulanic acid**	Oral	0.6	0.6	2.0	1.8	0.8	5.8
Mean relative change (%)			0	233.33	200	33.33	116.66
**Flucloxacillin**	Oral	0.2	0.2	0.3	0.2	0.6	1.5
Mean relative change (%)			0	50	0	200	62.50
**Benzathine benzyl penicillin**	IM	0.00002	0.0001	0.004	0.2	0.00001	0.2
Mean relative change (%)			400	19,900	999,900	-50	255,037.50
**Benzylpenicillin**	IM	0.0001	0	0	0.2	0	0.2
Mean relative change (%)			-100	-100	199,900	-100	49,900
Total		1,318.7	620.1	795.8	3,457.3	771.3	
Cephalosporins							
**Ceftriaxone**	IV/IM	0.1	3	7	65	176.7	251.8
Mean relative change (%)			2,900	6,900	64,900	176,600	62,825
**Cefixime**	Oral	0.01	0.1	0.1	0.4	1.6	2.2
Mean relative change (%)			900	900	3900	15900	5400
**Cefpodoxime**	Oral	0.003	0.01	0.03	0.04	0.05	0.13
Mean relative change (%)			233.33	900	1,233.33	1,566.66	983.33
**Cefotaxime**	IV/IM	0	0	0.003	0	0	0.003
Mean relative change (%)			0	_	-100	-100	-100
**Cefalexin**	Oral	0.04	0.07	0.1	0.06	0.2	0.47
Mean relative change (%)			75	150	50	400	168.75
**Cefadroxil**	Oral	0	0	0.02	0.02	0.02	0.06
Mean relative change (%)			0	–	0	0	0
Total		0.15	3.18	7.25	65.5	178.57	
Quinolones							
**Ciprofloxacin**	Oral	0.4	19.6	304.1	225.1	185.7	734.9
Mean relative change (%)			4,800	75,925	56,175	46,325	45,806.25
**Ofloxacin**	Oral	0.06	0.01	0.2	0.1	0.4	0.57
Mean relative change (%)			-83.33	233.33	66.67	566.67	195.83
**Levofloxacin**	Oral	0.004	0.05	0.01	0.08	0.6	0.74
Mean relative change (%)			1150	150	1900	14900	4525
**Norfloxacin**	Oral	0	0	0.01	0.02	0.12	0.13
Mean relative change (%)			0	_	100	1100	600
**Moxifloxacin**	Oral	0	7.1	0	0	0	7.1
Mean relative change (%)			–	-100	-100	-100	-100
Total		0.5	26.7	304.1	225.3	186.8	
Macrolides							
**Erythromycin**	Oral	187.5	125	11.8	187	27.8	539.1
Mean relative change (%)			-33.33	-93.71	-0.27	-85.17	-53.12
**Azithromycin**	Oral	0	7	0	62.3	14	83.3
Mean relative change (%)				-100	790	100	263.33
Total		187.5	132	11.8	249.3	41.8	
Sulfonamides, cyclines, and aminoglycosides							
**Sulfamethoxazole/trimethoprim (cotrimoxazole)**	Oral	124.7	270.3	365.1	10.5	250.4	1021
Mean relative change (%)			116.76	192.78	-91.58	100.80	79.69
**Doxycycline**	Oral	561	190	0	0	20.5	771.5
Mean relative change (%)			-66.13	-100	-100	-96.34	-90.62
**Gentamycin**	IV/IM	0	0	0	0	64	64
Mean relative change (%)		–	–	–	–	–	
Total		685.7	460.3	365.1	10.5	334.9	

**Table 3:** Classification of antibiotics according to their level of consumption in DDDs per 1,000 inhabitants/year and their AWaRe group.

N	Antibiotics	DDD by 1,000 inhabitants/year	AWaRe classification
1	Amoxicillin	1023.26	Access group
2	Ampicillin	248.6	Access group
3	Sulfamethoxazole/trimethoprim	204.2	Access group
4	Doxycyclin	154.3	Access group
5	Ciprofloxacin	146.98	Watch group
6	Phénoxyméthyl-pénicillin	119.24	Access group
7	Erythromicin	107.82	Access group
8	Ceftriaxone	50.36	Watch group
9	Azithromycin	16.66	Watch group
10	Gentamicin	12.8	Access group
11	Moxifloxacin	1.42	Watch group
12	Amoxicillin + clavulanic acid	1.16	Access group
13	Cefixime	0.44	Watch group
14	Flucloxacillin	0.3	Access group
15	Levofloxacin	0.148	Watch group
16	Ofloxacin	0.114	Watch group
17	Cefalexine	0.094	Watch group
18	Benzathine benzyl penicillin	0.04	Access group
19	Benzylpenicillin	0.04	Access group
20	Cefpodoxime	0.026	Watch group
21	Norfloxacin	0.026	Watch group
22	Cefadroxil	0.012	Watch group
23	Cefotaxime	0.0006	Watch group

### 3.2 Daily dose of ABC per 1,000 in habitants/year

**Figure 5:**
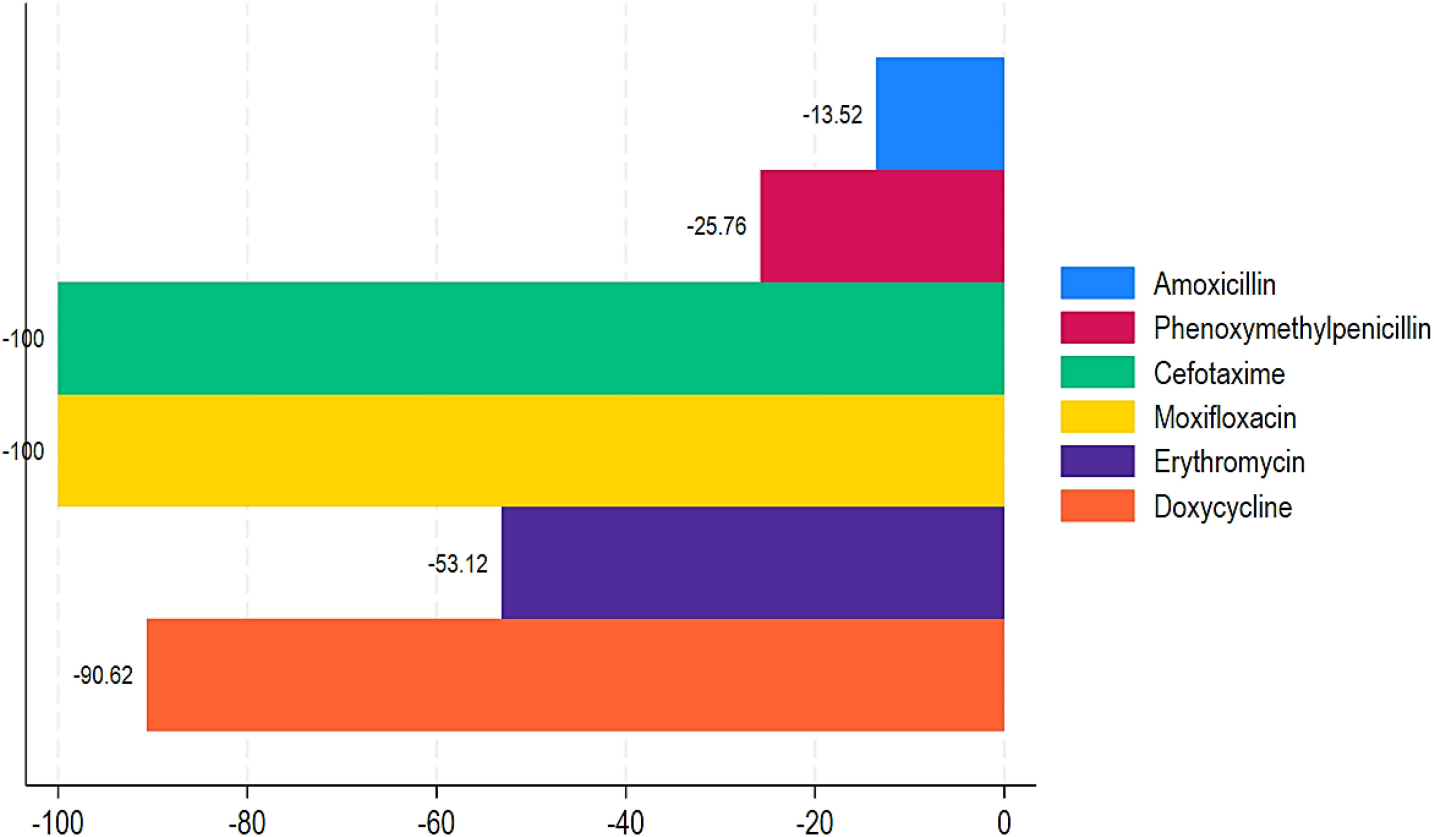
Proportion of negative mean relative change in ABC in Chad (2017–2021).

**Figure 5** depicts the total ABC of DDDs using imports and wholesalers’ data. Except for 2017 and 2020, ABC was similar in the data from both sources. Between 2017 and 2021, each resident would have consumed 1.5 to 5 doses of antibiotics, with an average of 2.5 doses per year, peaking in 2020 at 5.4 doses per inhabitant (**Figure 5**).

**Table 1** shows the trend of ABC by DDDs per 1,000 inhabitants and MRC (%) from 2017 to 2021. During this time period, aminoglycosides, other beta-lactam antibacterials, quinolones, and combinations of antibacterials showed highly increased ABC with MRCs of 290,920%, 29,112.5%, 36,245%, and 5,400%, respectively (**Table 1**; **Figure 2**). Similarly, penicillin and sulfonamides and trimethoprim showed a relatively increased MRC of 28.82% and 72.12%, respectively. In contrast, tetracyclines, chloramphenicols, macrolides, and other antibacterials showed decreased ABC of -90.62%, -70.73%, -48.53%, and -34.71% (**Table 1**; **Figure 3**).

### 3.3 ABC of various antibiotics expressed in DDDs per 1,000 inhabitants/year

**Table 2** displays the ABC in DDDs per 1,000 inhabitants/year by international common name (INN). Amoxicillin (1,023.3 doses per 1,000 inhabitants/year), ampicillin (248.6), sulfamethoxazole/trimethoprim (204.2), doxycycline (154.3), ciprofloxacin (147), phenoxymethyl-penicillin (119.2), erythromycin (107.8), ceftriaxone (50.36), azithromycin (16.7), and gentamicin (12.8) were the 10 most commonly used antibiotics between 2017 and 2021. During the study period, each individual would have taken five doses of amoxicillin, one per year, and 2.5 in 2020.

Our results also demonstrated that among the penicillins, benzathine, benzyl penicillin, benzylpenicillin, and ampicillin showed a substantial increase in ABC with MRCs of 255,037.50%, 49,900%, and 1,405.79% from 2017 to 2021 (**Table 2**; **Figure 4**). Similarly, amoxicillin + clavulanic acid and flucloxacillin showed an increased ABC with MRCs of 116.66% and 62.50% (**Table 2**, **Figure 4**). Regarding the ABC trend of cephalosporins, all of them showed an increased MRC, except cefotaxime (MRC: -100%). Ceftriaxone had the greatest MRC (62,825%), followed by cefixime (MRC: 5,400%), cefpodoxime (MRC: 983.33%), and cefalexin (168.75%) (**Table 2**; **Figure 4**). For the quinolones, ciprofloxacin, levofloxacin, norfloxacin, and ofloxacin showed high MRC with 45,806.25%, 4,525%, 600%, and 195.83%, respectively (**Table 2**; **Figure 4**). In contrast, moxifloxacin showed a decrease of -100% (**Table 2**; **Figure 5**). For the macrolides, azithromycin had an increase in MRC of 263.33% (**Figure 4**). Conversely, erythromycin had a reduction of -53.12% (**Figure 5**). Lastly, our findings showed an increased MRC for cotrimoxazole (79.69%) (**Figure 4**) and a decreased MRC for doxycycline (-90.62%) (**Figure 4**). Interestingly, a DDD for gentamycin was only recorded in 2021, with 64 (**Table 2**).

### 3.4 Comparison of ABC data with the WHO recommendations for usage via the AWaRe classification of antibiotics

**Figure 6:**
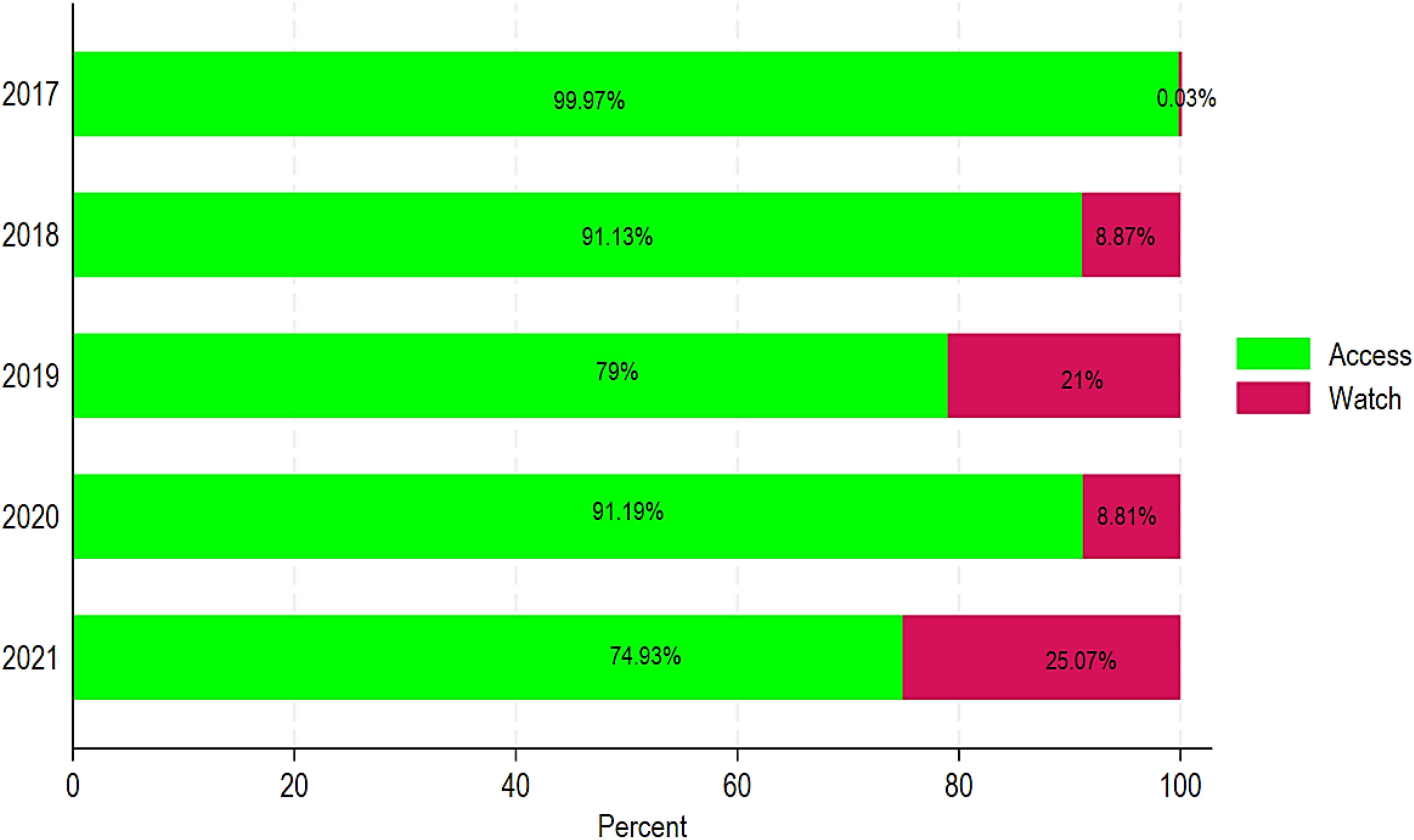
Relative ABC by Access and Watch classification in Chad from 2017 to 2021.

ABC was classified into two groups under the AWaRe classification: Access and Watch. When compared to the AWaRe categorization, three antibiotics from the “Watch” group rank in the top ten most consumed antibiotics: ciprofloxacin, ceftriaxone, and azithromycin (**Table 3**). Furthermore, 90% of the antibiotic doses consumed throughout the period were of antibiotics in the “Access” group (**Table 3**). When comparing ABC in DDDs per 1,000 inhabitants/year by Access vs. Watch, our results showed 99.97% vs. 0.03% in 2017, 91.13% vs. 8.87% in 2018, 79% vs. 21% in 2019, 91.19% vs. 8.81% in 2020, and 74.93% vs. 25.07% in 2021 (**Figure 6**).

## 4 Discussion

The WHO proposed a global monitoring indicator requiring that all ABC come from the “Access” group, including antibiotics with the lowest risk of resistance (Klein et al., 2021). This study’s aim was to quantify, track, and describe ABC data for the import and wholesale data in Chad from 2017 to 2021 and to report on it using the WHO AWaRe classification. Our results showed a high ABC in the Chadian population from 2017 to 2021 based on import and wholesale data. The DDD and MRC showed increased ABC of mainly penicillin (benzathine benzyl penicillin, benzylpenicillin, ampicillin, amoxicillin + clavulanic acid, flucloxacillin, ceftriaxone, cefixime, cefpodoxime, and cefalexin), sulfonamides/trimethoprim (cotrimoxazole), aminoglycosides, other beta-lactam antibacterials, quinolones (ciprofloxacin, levofloxacin, norfloxacin, and ofloxacin), and antibacterial combinations. In contrast, tetracyclines (doxycycline), chloramphenicols, macrolides (erythromycin), and other antibacterials had a decreased MRC. Our findings also demonstrated that the trend of Access vs. Watch group usage has increased over time in Chad. In comparison with other studies conducted in SSA, ABC in DDDs per 1,000 inhabitants/year and the MRC were higher in Chad (Mbwasi et al., 2020; Kanu et al., 2021; Namugambe et al., 2021; Mthombeni et al., 2022). This could explain the reasons for Chad’s high ABR, as several studies have documented (Ndoutamia et al., 2014; Nadlaou et al., 2015; Cardinale et al., 2020; Ahmat et al., 2023). High MRC in aminoglycosides indicated that they were used less in prior years, but increased dramatically in subsequent years. Aminoglycosides are widely used in SSA due to their effectiveness against Gram-negative bacilli infections and availability in some countries. This has a high impact on ABR associated with mortality in Chad. A report indicated that there were 5,800 deaths due to ABR and a total of 25,100 deaths in Chad in 2019, positioning Chad among the top 10 countries in age-standardized mortality rate per 100,000 population related to ABR across 204 countries (Institute for Health Metrics and Evaluation, 2024). In Chad, the mortality associated with ABR was mainly associated with five pathogens, including *Streptococcus pneumoniae*, *Klebsiella pneumoniae*, *Escherichia coli*, *Group B Streptococcus*, and *Staphylococcus aureus* (Institute for Health Metrics and Evaluation, 2024). This calls for immediate action on the monitoring system for ABC at all levels in Chad, as shown in **Figure 1**. However, in recent years, there has been minimal global progress on the monitoring system for ABC (WHO, 2022). Establishing an effective national monitoring system for antimicrobial use/consumption in humans is crucial for monitoring data on the prescription, import, wholesale, sale, and use of antimicrobials, as well as legislative enforcement (WHO, 2022).

ABC showed an increasing trend in Chad between 2017 and 2021, with a peak in 2020, linked to the COVID-19 pandemic. The COVID-19 pandemic led to a rise in ABC for antibiotics such as azithromycin and ceftriaxone. Approximately half of the patients hospitalized for COVID-19 were given ceftriaxone, which was frequently combined with azithromycin (CDC, 2021). Furthermore, another analysis revealed that the COVID-19 pandemic caused global alterations in antibiotic prescribing trends, with heightened ABC observed in various nations, possibly due to concerns about secondary infections in patients with COVID-19 (Klein et al., 2021). A study proposed that population-level ABC surveillance is critical for overseeing and regulating ABC during and after pandemics like the COVID-19 pandemic (Van Boeckel et al., 2019). A significant increase in ABC has also been observed in other pandemic contexts, where the misuse of antibiotics has been reported due to the lack of specific treatments in Africa (Egyir et al., 2020; Piddock et al., 2022; WHO, 2024b). In Europe and America, a decrease in ABC was noted during the same period, but this was linked to containment measures and the reduction in medical consultations (WHO, 2024b). Studies have shown that the high consumption of penicillins, linked to respiratory tract infections, is consistent with trends observed in many countries, where respiratory infections remain the leading cause of antibiotic use (van Houten et al., 2019; Mustafa et al., 2023). However, the high use of certain antibiotics, such as penicillins and other beta-lactams, requires special attention because this could contribute to ABR, particularly to infections caused by *Streptococcus pneumoniae*, *Haemophilus influenzae*, and *Escherichia coli* (Cillóniz et al., 2018; Tadele et al., 2019). Among the penicillins, our study showed a high increase in the MRC in the ABC of benzathine benzylpenicillin, benzylpenicillin, and ampicillin. Similarly, amoxicillin + clavulanic acid and flucloxacillin showed a moderate increase in the MRC in the ABC. The trends in the import and wholesale data also showed an increased ABC of aminoglycosides, other beta-lactam antibacterials, quinolones, and antibacterial combinations in terms of DDDs per 1,000 inhabitants/year and MRC from 2017 to 2021. Our results revealed that aminoglycosides, other beta-lactam antibacterials, quinolones, and antibacterial combinations showed high increased ABC trends, including ciprofloxacin, levofloxacin, norfloxacin, and ofloxacin. The increased use of quinolones has led to increasing resistance to these antimicrobials, with rates of resistance that vary by both organism and geographic region (Jacoby, 2005). A study has shown that commonly used quinolones such as levofloxacin and ciprofloxacin have lower response rates against Gram-negative bacteria, including *E. coli, K. pneumoniae*, and *P. aeruginosa*, compared with other antibiotics used in the ICUs (DeRyke et al., 2007; Kherroubi et al., 2024). The ABR rate of *E. coli* to amoxicillin, trimethoprim, and gentamicin was 88.1%, 80.7%, and 29.8%, respectively (Tadesse et al., 2017). *Neisseria gonorrhoeae* was reported to have an antimicrobial resistance (AMR) rate to quinolone of 37.5% (Tadesse et al., 2017). Carbapenem resistance was common in *Acinetobacter* spp. and *Pseudomonas aeruginosa* (Tadesse et al., 2017). This group includes the majority of the bacteria associated with ABR and mortality in Chad.

Our results also showed that 90% of doses consumed were in the “Access” group. Thus, the consumption profile in Chad far exceeds the WHO target, which recommends that 60% of antibiotics belong to this group to minimize the risk of resistance. Furthermore, our results showed that the “Watch” group use increased over time in Chad. Although antibiotics in the “Access” group are less likely to cause resistance when used correctly, overuse can still generate selective pressure on bacteria, leading to the selection of resistant strains (Costelloe et al., 2010). “Watch” group antibiotics, such as ciprofloxacin, ceftriaxone, and azithromycin, are of particular concern because their overuse is associated with an increased risk of ABR (Zanichelli et al., 2023). A study showed that the use of “Watch” antibiotics was related to roughly twice the risk of subsequent colonization or infection with multidrug-resistant bacteria than not having been exposed to “Watch” medicines (Zanichelli et al., 2023). Exposure to third-generation cephalosporins raised the risk of developing Enterobacterales, which produce extended-spectrum β-lactamases. Similarly, quinolone exposure increased the risk of methicillin-resistant *Staphylococcus aureus*, and carbapenem exposure increased the risk of carbapenem-resistant *Acinetobacter baumannii*, Enterobacterales, and *Pseudomonas aeruginosa* (Zanichelli et al., 2023). These findings support the WHO AWaRe antibiotic guidelines, which aim to increase the proportion of patients treated with “Access” antibiotics or no antibiotic treatment if possible (Zanichelli et al., 2023). The “Watch” group antibiotics, although effective, should be reserved for specific indications to avoid their inappropriate use and should be used with caution (Pulcini, 2017; WHO, 2018). Our results revealed that there is ABC of both groups of the AWaRe classification, namely, the “Access” and “Watch” groups. When comparing the level of consumption with the AWaRe classification, three antibiotics from the “Watch” group were among the 10 most consumed antibiotics: ciprofloxacin, ceftriaxone, and azithromycin. An escalation in the “Watch” group ABC is a significant threat in Chad and may elucidate the country’s elevated ABR. In addition, roughly 90% of the doses of ABC during the study period were antibiotics from the “Access” group, which, according to the AWaRe recommendations, should be used as first- or second- line treatment.

Based on the above discussion, Chad should effectively and urgently implement its national strategic plan for the fight against ABR (Ministere de la Sante Publique, 2018). This strategic plan includes five strategic axes, including enhancing awareness and comprehension of ABC, establishing surveillance and research initiatives, decreasing infection rates through effective preventive measures (hygiene, vaccination) in human health sectors, optimizing the utilization of antimicrobial agents in human health, and strengthening the regulatory framework to combat ABR (Ministere de la Sante Publique, 2018). Furthermore, novel evidence-based strategies and technologies should be explored to improve these five axes. Recent studies have shown that digital tools can facilitate the early diagnosis of bacterial infectious diseases, fulfilling the necessity for precise diagnoses to avert delays in infection identification, inadequate risk stratification, and a limited comprehension of local ABR trends (Health, 2024). Nonetheless, numerous LMICs such as Chad, encounter obstacles including limited infrastructure, insufficient investment and human resources, underutilization of accessible health-care data, and ineffective communication of information to regulatory authorities (Health, 2024). The mapping ABR and ABC partnership reports that merely 1.3% of the 50,000 medical laboratories in the networks of the 14 participating African countries perform bacteriology testing (Health, 2024). Digital tools, including computerized decision support systems, mobile health applications, and wearable devices, can play a pivotal role in the implementation of effective and cost-efficient diagnostic strategies and advances for ABR. Tan et al. showed in a cluster randomized controlled trial in Tanzania that a digital clinical decision support tool with diagnostic tests and mentorship reduced antibiotic prescriptions to 23.2% compared with 70.1% in the usual care cluster, without compromising patient safety (Health, 2024; Tan et al., 2024). In a randomized controlled trial with 249 participants, Plechatá et al. showed that experiential virtual reality significantly increased the intention to use antibiotics prudently more effectively than informational virtual reality or leaflet information (Health, 2024; Plechatá et al., 2024).

This is the first study reviewing nationwide data related to ABC in Chad as recommended by the WHO. This study recommends establishing a baseline for ABC in Chad through policy, practice, and research initiatives. The study’s findings indicate the need for increased antibiotic-awareness efforts, stricter enforcement of prescription-only rules, and prudent prescribing procedures in Chad. This study also recommends that the national ABR action plan (Ministere de la Sante Publique, 2018) that has been developed in Chad should be implemented effectively in the short term, and this data should be used to ensure this progression. The study’s limitations included bias due to the refusal of the most viable private wholesalers to participate. This may have caused sampling bias. However, the wholesalers involved in the study are the primary market participants, as evidenced by the distribution volume in the country. The data gathered from these wholesalers is akin to import data; however, it may not accurately reflect real ABC by the end user, presenting a potential bias. Numerous studies suggest that ABC figures derived from imports and distribution may inflate the true ABC due to insufficient oversight of their dispensation (Goossens et al., 2005; Klein et al., 2021). In addition, the study does not take into account parallel and illicit circuits, which can have a significant impact on the availability of ABC in low-resource countries (Laxminarayan et al., 2013). “Reserve” group antibiotics, which should only be used for specific indications such as infections with multidrug-resistant bacteria, account for less than 2% of total antibiotic consumption in most high-income countries (World Health Organization, 2018). However, our study did not report on “Reserve group” antibiotics. This should be considered one of the study’s limitations. Another limitation is the fact that this study depends on import and wholesale data, which may not correctly represent actual consumption due to possible stockpiling, outdated drugs, or diversion to informal markets. Because this analysis was based on aggregate DDDs rather than prescription or patient-level data, it provided limited insights into real usage patterns such as informal prescribing and self-medication. In Chad, ABC may vary both seasonally and regionally, and be affected by seasonal infectious disease epidemics and geographical variances in healthcare access and antibiotic management. However, seasonal or geographical changes in ABC were not included in this study. Future research should focus on seasonal or regional differences that cause epidemics such as measles, gastroenteritis, respiratory tract infections, and ABC in Chad. Studies involving prescription or patient-level data, informal prescribing, and self-medication are also required to show the overall ABC in Chad.

## 5 Conclusion

This study provides an initial analysis of ABC in Chad based on import and distribution data from major wholesalers. Our results showed high ABC in the population in Chad from 2017 to 2021, which could explain the high ABR in the country. Furthermore, our study showed that an average of 90% of doses consumed were antibiotics in the “Access” group and there was increased use of antibiotics in the “Watch” group over time, which are more likely to increase ABR. This calls for immediate action on the monitoring system for ABC at all levels in Chad. Particular attention should be paid to the use of antibiotics in the “Watch” group and to the fight against self-medication, which remains an important factor in the mismanagement of antimicrobials. The results highlight the need for stricter regulation of the pharmaceutical market in Chad.

## Data Availability

The raw data supporting the conclusions of this article will be made available by the authors, without undue reservation.
