# Supplementary material for: National antibiotic consumption for human use in Chad (2017–2021): a descriptive cross-sectional study
Source: Front Antibiot. 2025 Jul 11;4:1612557. doi: 10.3389/frabi.2025.1612557 (PMC12290472; doi:10.3389/frabi.2025.1612557)
Supplement: Supplementary file 1 [file Supplementaryfile1.docx]

Supplemental Material: **National Antibiotic Consumption for Human Use in Chad (2017–2021): A Descriptive Cross-Sectional Study**

Supplemental Table 1: Number of commercial and common designations of antibiotics recorded

| **Year** | **Import data** | **Wholesale Data** |
| --- | --- | --- |
| **Number of commercial antibiotic designs evaluated** | | |
| 2017 | 110 | 110 |
| 2018 | 130 | 131 |
| 2019 | 133 | 134 |
| 2020 | 151 | 151 |
| 2021 | 168 | 169 |
| **Number of common international antibiotic designations reviewed** | | |
| 2017 | 26 | 26 |
| 2018 | 28 | 29 |
| 2019 | 35 | 36 |
| 2020 | 34 | 34 |
| 2021 | 37 | 38 |

Supplemental Table 2: Defined Daily Dose of ABC by pharmacological group from wholesale data

| Code ATC3 | Pharmacological group | 2017 | 2018 | 2019 | 2020 | 2021 | Total /Mean Relative Change (%) |
| --- | --- | --- | --- | --- | --- | --- | --- |
| J01C | **Penicillins** | 19504079 | 25153605 | 12505838 | 57860932 | 12987717 | 128012171 |
| \| Mean Relative Change (%) \| \| --- \| | |  | 28.96 | -35.88 | 196.66 | -33.41 | 39.08 |
| J01D | **Other beta-lactam antibacterials** | 2079 | 47959 | 115888 | 1059885 | 2987841 | 4213652 |
| Mean Relative Change (%) | |  | 2206.83 | 5474.22 | 50880.52 | 143615.29 | 50544.21 |
| J01M | **Quinolones** | 7200 | 301277 | 4782092 | 3647900 | 3118338 | 11856807 |
| Mean Relative Change (%) | |  | 4084.40 | 66317.94 | 50565.28 | 43210.25 | 41044.47 |
| J01F | **Macrolides** | 2994897 | 1954691 | 98459 | 3833027 | 761756 | 9642830 |
| Mean Relative Change (%) | |  | -34.73 | -96.71 | 27.98 | -74.56 | -44.50 |
| J01X | **Other antibacterials** | 1187145 | 1920812 | 128265 | 1189875 | 38538 | 4464635 |
| Mean Relative Change (%) | |  | 61.80 | -89.19 | 0.23 | -96.75 | -30.98 |
| J01R | **Antibacterial combinations** | 280 | 7295.65 | 11599 | 11542 | 42179 | 65600 |
| Mean Relative Change (%) | |  | 2505.59 | 4042.5 | 4022.14 | 14963.93 | 6383.54 |
| J01A | **Tetracyclines** | 8633600 | 3240544 | 22196 | 11560 | 60880 | 11968780 |
| Mean Relative Change (%) | |  | -62.46 | -99.74 | -99.87 | -99.29 | -90.94 |
| J01B | **Choramphenicols** | 182225 | 167474 | 31546 | 10522 | 10942 | 402709 |
| Mean Relative Change (%) | |  | -8.09 | -82.69 | -94.22 | -93.99 | -69.75 |
| J01E | **Sulfonamides and trimethoprim** | 2092500 | 4658704 | 6083669 | 277000 | 4423200 | 17535073 |
| Mean Relative Change (%) | |  | 122.64 | 190.74 | -86.76 | 111.38 | 84.50 |
| J01G | **Aminoglycosides** | 67 | 67 | 20837 | 27500 | 922491 | 970962 |
| Mean Relative Change (%) | |  | 0 | 31000 | 40944.78 | 1376752.24 | 362174.25 |
| Total |  | 34 604 073 | 37 452 431 | 23 800 393 | 67 929 744 | 25 353 885 |  |

Supplemental Table 3: Defined Daily Dose of ABC by pharmacological group from import data

| Code ATC3 | Groupe pharmacologique | 2017 | 2018 | 2019 | 2020 | 2021 | Total |
| --- | --- | --- | --- | --- | --- | --- | --- |
| J01C | Penicillines | 19504079 | 25153605 | 12505838 | 57860932 | 12987717 | 128012171 |
| \| Mean Relative Change (%) \| \| --- \| | |  | 28.96 | -35.88 | 196.66 | -33.41 | 39.08 |
| J01D | Autres antibactériens bêtalactamines | 2079 | 47959 | 115838 | 1059885 | 2987841 | 4213602 |
| Mean Relative Change (%) | |  | 2206.83 | 5474.22 | 50880.52 | 143615.29 | 50544.21 |
| J01M | Quinolones | 7200 | 193277 | 4782092 | 3647900 | 3118338 | 11748807 |
| Mean Relative Change (%) | |  | 4084.40 | 66317.94 | 50565.28 | 43210.25 | 41044.47 |
| J01F | Macrolides | 2994897 | 1954691 | 98459 | 3833027 | 761756 | 9642830 |
| Mean Relative Change (%) | |  | -34.73 | -96.71 | 27.98 | -74.56 | -44.50 |
| J01X | Autres antibactériens | 1187145 | 1920812 | 128265 | 1189875 | 32653 | 4458750 |
| Mean Relative Change (%) | |  | 61.80 | -89.19 | 0.23 | -97.25 | 31.10 |
| J01R | Combinaison d’antibactérien | 280 | 7295 | 11599 | 11542 | 42179 | 72895 |
| Mean Relative Change (%) | |  | 2505.36 | 4042.5 | 4022.14 | 14963.93 | 6383.48 |
| J01A | Tétracycline | 8633600 | 3240544 | 22196 | 11560 | 60880 | 11968780 |
| Mean Relative Change (%) | |  | -62.46 | -99.74 | -99.87 | -99.29 | -90.94 |
| J01B | Amphénicols | 182225 | 167474 | 31546.66 | 10522.66 | 10943 | 360642 |
| Mean Relative Change (%) | |  | -8.09 | -82.69 | -94.22 | -93.99 | -69.75 |
| J01E | Sulfonamides et triméthoprime | 2092500 | 4658704 | 6083669.2 | 277000 | 4423200 | 11451404 |
|  | |  | 122.64 | 190.74 | -86.76 | 111.38 | 84.5 |
| J01G | Aminoglycosides | 67 | 67 | 20838 | 27500 | 922492 | 970964 |
| Mean Relative Change (%) | |  | 0 | 31001.49 | 40944.78 | 1376753.73 | 362175 |
| Total |  | 34604073 | 37344431 | 23800343 | 67929745 | 25348001 |  |


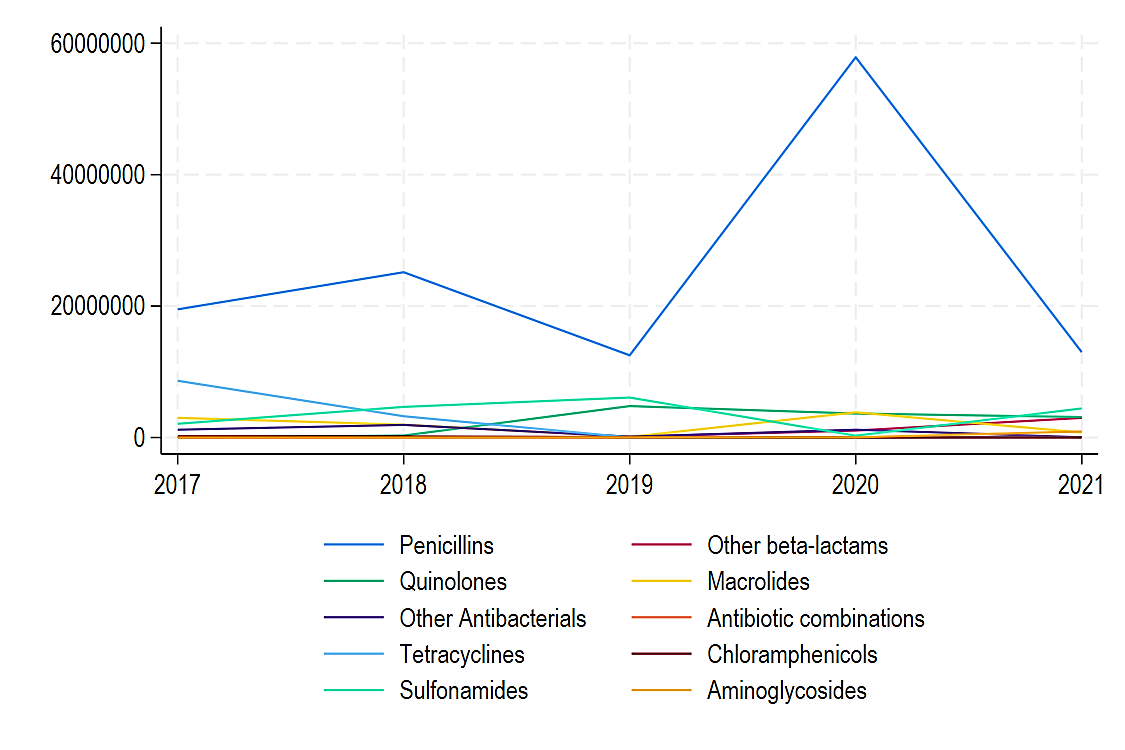


**Supplementa Figure 1: Trend of approximate mean DDD from** **wholesale and import data in Chad from 2017-2021.**


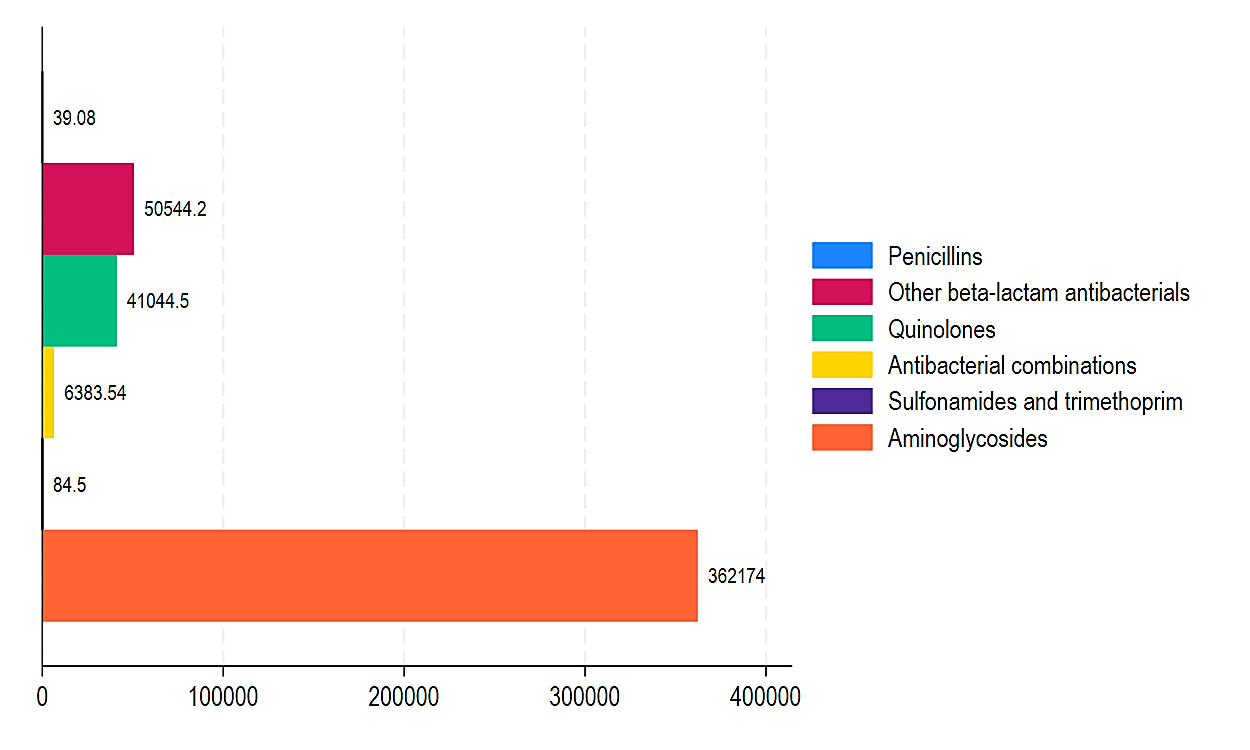


**Supplemental Figure 2:** Proportion of positive mean relative change of antibiotic groups consumed in Chad (2017-2021)


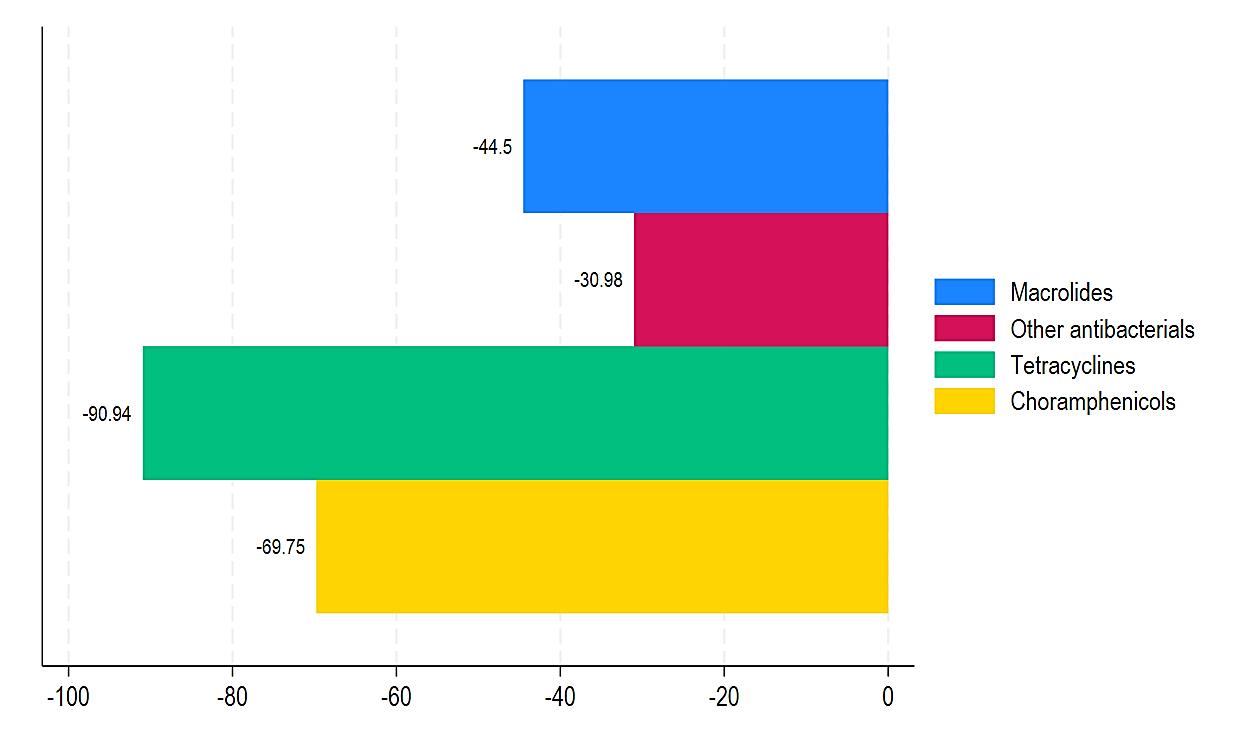


**Supplemental Figure 3:** Proportion of negative mean relative change of antibiotic groups consumed in Chad (2017-2021)


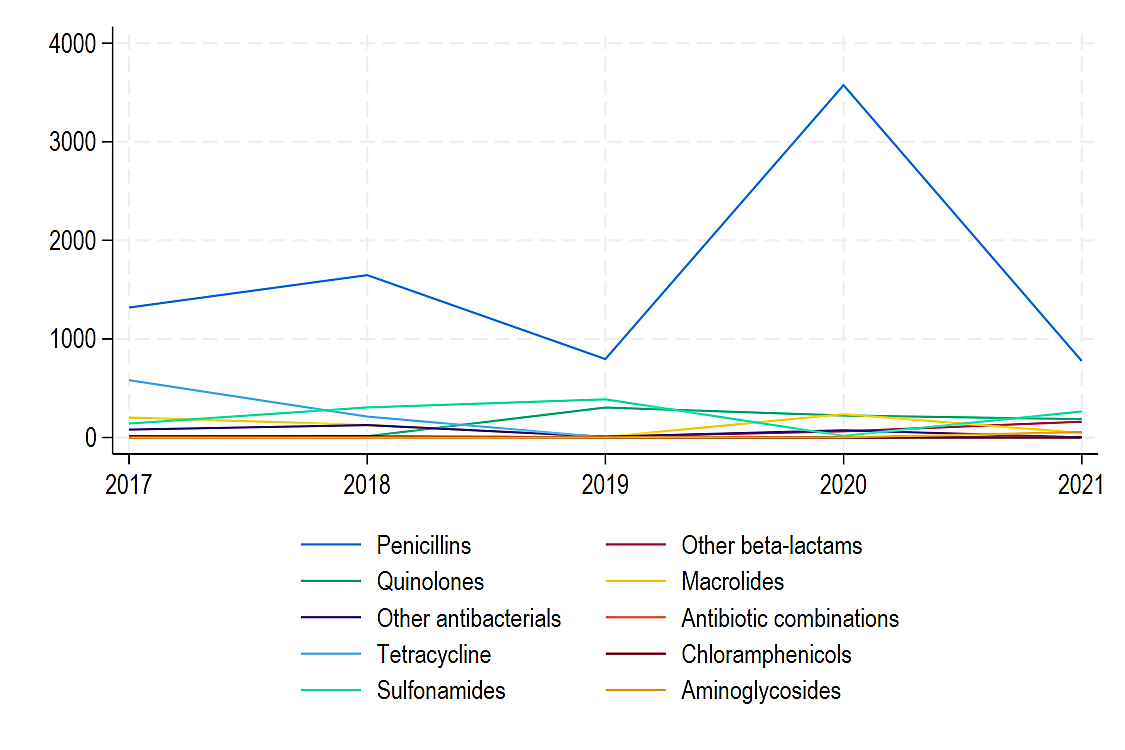


**Figure 4: Trend of approximate mean DDD in 1000 habitant annually in Chad from 2017-2021.**
